# Association of Central Precocious Puberty with a Rare Presentation of Schimmelpenning–Feuerstein–Mims Syndrome in a Peruvian Girl

**DOI:** 10.1155/2020/1928121

**Published:** 2020-07-09

**Authors:** Miguel Angel De los Santos-La Torre, Carlos Manuel Del Águila-Villar, Luis Rómulo Lu-de Lama, Oswaldo Nuñez-Almache, Eliana Manuela Chávez-Tejada, Oscar Antonio Espinoza-Robles, Paola Marianella Pinto-Ibárcena, Martha Rosario Calagua-Quispe

**Affiliations:** ^1^Department of Endocrinology and Metabolism, The Child's Health National Institute (Instituto Nacional de Salud del Niño), Lima, Peru; ^2^Faculty Member of the Medical School, Universidad Nacional Federico Villareal, Lima, Peru

## Abstract

Schimmelpenning–Feuerstein–Mims syndrome (SFM), an epidermal nevus syndrome characterized by skin lesions, has an estimated incidence of 1 per 10 000 live births. Nevus sebaceous, the most common cutaneous lesion, and verrucous nevus, the less frequent lesion, are coupled with a wide range of extracutaneous manifestations. As part of these manifestations, rarely, central precocious puberty can arise. We report the case of a 1-year-5-month-old girl who presented to the Endocrinology and Metabolism Department with breast enlargement that began at one year of age, growth of pubic and axillary hair three months later, and vaginal bleeding that occurred five months later. During clinical examination, melanocytic nevi, with a diameter ranging from 3 to 5 mm, were noted on the face. Verrucous nevi of variable size with a tendency for coalescence following the lines of Blaschko and melanocytic nevi with a diameter ranging from 3 to 6 mm were observed on the right hemibody and on the left hemibody, respectively. Right asymmetry of the lower extremities was observed. Laboratory findings showed a significant increase in the levels of follicle-stimulating hormone (FSH) and luteinizing hormone (LH) after the gonadotropin-releasing hormone (GnRH) stimulation test; additionally, imaging demonstrated advanced bone age and pubertal changes in the internal genitals. Analyses of the H-RAS, K-RAS, and N-RAS genes in the blood and in the skin were performed, and a missense mutation in exon 2 of the gene, H-RAS c37G > C (p.G13R), was detected in the latter. Treatment with triptorelin, a GnRH analog, was initiated, and it gave good clinical response. Epidermal nevus syndrome has a wide and variable systemic involvement. Thus, it is important to consider the development of precocious puberty for a prompt diagnosis and to strategize a multidisciplinary approach from the beginning.

## 1. Introduction

Schimmelpenning–Feuerstein–Mims (SFM) syndrome, an epidermal nevus syndrome characterized by skin lesions, has an estimated incidence of 1 per 10 000 live births [1]. Nevus sebaceous, the most common cutaneous lesion, and verrucous nevus, the less frequent lesion, occur in conjunction with several organic and systemic anomalies such as neurological, skeletal, cardiovascular, ophthalmic, and urologic disorders [2–4]. As part of these, endocrine abnormalities, such as hypophosphatemic vitamin D resistant rickets, syndrome of inappropriate antidiuretic hormone (SIADH), and more rarely central precocious puberty (CPP), are manifested; however, very few cases have been reported so far [5, 6].

The syndrome was first described by Josef Jadassohn in 1895 [5, 7]. Several years later, Gustav Wilhelm Schimmelpenning, in 1957, described the main clinical features of the syndrome in a 17-year-old adolescent and proposed it is a new phacomatosis [2]. Later, Richard C. Feuerstein and Leroy C. Mims described two cases of linear epidemic nevus with seizures and mental retardation in 1962, while Laurence Solomon, in 1968, made important contributions in expanding the characterization of the dermal component [2].

SFM syndrome is one of nine different types of epidermal nevus syndromes [7]. It has a sporadic presentation, and so far, familial cases with SFM syndrome have not been reported. Furthermore, a discordance between monochorionic twins, where one twin has the syndrome and the other is completely healthy, has been reported in both males and females [8]. The cause of the syndrome is still unknown, although Happle's hypothesis is the one that has gained the most acceptance. According to Happle, SFM syndrome would result from a genomic mosaicism where the mutated cell, as a result of a postzygotic mutation in early embryogenesis, survives only in close proximity to healthy cells. Current reports reveal that such a mutation would occur in any of the following three genes: H-RAS [9–11], K-RAS [9], and N-RAS [12]. The distribution of the skin lesions following the Blaschko lines (lines on the skin that represent the pattern of growth and development during epidermal cell migration), an important feature of the syndrome, could be explained by this hypothesis [13, 14].

The first association of SFM syndrome with CPP was described by Moss et al. in a 2-year-4 month-old boy with extensive verrucous nevi and secondary sexual characteristics [15]. Other cases with CPP have also been identified in Hong Kong [16], the United States [17, 18], Germany [19], Iran [20], and India [21]. It has been proposed that extensive verrucous nevi release factors that would lead to a premature activation of the gonadotropic axis in these patients [18, 21].

Herein, we report a case of CPP and an atypical presentation of SFM syndrome in a girl. This is the first Peruvian case and the eighth case overall, showing an association between CPP and SFM syndrome. Written informed consent was obtained for the photographs presented in this case report.

## 2. Case Presentation

A 1-year-5-month-old girl presented to the Endocrinology and Metabolism Department with breast enlargement that began at one year of age, pubic hair and axillary hair that appeared 3 months later, and vaginal bleeding that occurred 5 months after the onset of breast development. She was the first-born child, born at 40 weeks, weighing 3.540 kg, and a length of 51 cm. Since birth, melanocytic and verrucous nevi were noted over her face, trunk, and extremities. Her psychomotor development was normal. There was no consanguinity between the parents.

**Figure 1:**
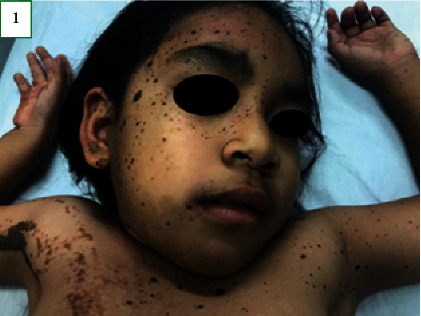
Skin patterns of the melanocytic and verrucous nevi on the face and the upper trunk

**Figure 2:**
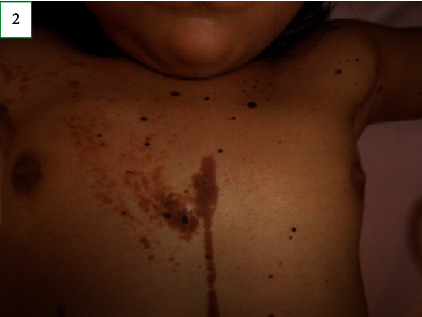
Breast development.

**Figure 3:**
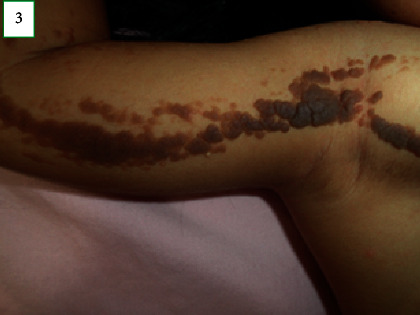
Skin patterns of the melanocytic and verrucous nevi.

**Figure 4:**
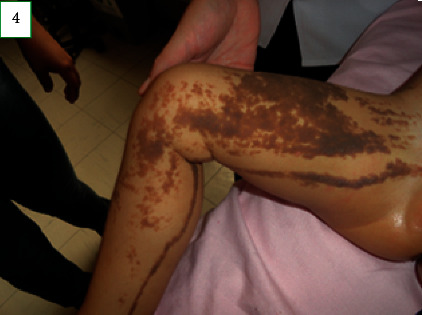
Skin patterns of the verrucous nevi on the right lower extremity

On physical examination, the child's weight and head circumference were at the 75^th^ percentile for her age. Her length was at the 90^th^ percentile. Skin examination revealed melanocytic nevi with a diameter ranging from 3 to 5 mm on the face (Figure 1). Verrucous nevi of variable size with a tendency for coalescence following the lines of Blaschko, stopping at the midline, were observed on the right hemibody. Furthermore, melanocytic nevi, with a diameter ranging from 3 to 6 mm, were observed on the left hemibody (Figures 2–4). According to the Tanner stage pubertal development, the patient had breast stage IV, pubic hair stage III, and axillary hair Wolfsdorf stage II. Right asymmetry of the lower extremities was noted during the examination.

**Figure 5:**
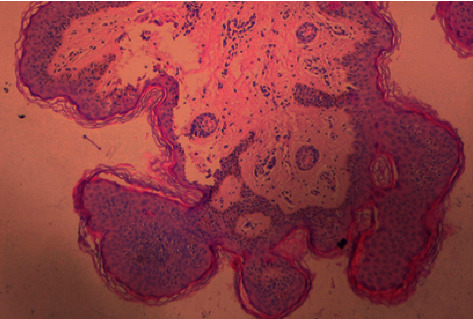
Skin biopsy: histological features of right skin lesions showed papillomatosis, acanthosis, and hyperkeratosis, which were consistent with verrucous epidermal nevus.

Pelvic ultrasound revealed the following: uterine size and volume, 3.75 cm × 2.4 × 1.4 cm and 6 mL, respectively; endometrium size, 5 mm; right ovarian volume, 2.8 mL; left ovarian volume, 3.3 mL; and ovarian follicles with diameter >5 mm. Bone age was 4.0 years old. Laboratory values showed a significant increase in the levels of follicle-stimulating hormone (FSH) and luteinizing hormone (LH) after the GnRH stimulation test. The results are as follows: basal LH: 3.3 IU/L and 60 min LH: 72.1 IUI/L; basal FSH: 3.9 IU/L and 60 min FSH: 18.8 IU/L. Skin biopsy, from the right hemibody lesions, showed histological features of papillomatosis, acanthosis, and hyperkeratosis, which were consistent with verrucous epidermal nevus (Figure 5). Laboratory investigation demonstrated normal levels of serum calcium, phosphorus, urinary phosphate, electrolytes, parathyroid hormone (PTH), and vitamin D. Renal and adrenal ultrasound, skeletal survey, and brain MRI showed no abnormalities.

Analyses of the H-RAS, K-RAS, and N-RAS genes in the blood and in the skin were performed, and in the latter, a missense mutation in exon 2 of the gene, H-RAS c37G > C (p.G13R), was detected. The DNA was extracted and purified using DNA binding columns (Qiagen). DNA integrity and quality were evaluated with spectrophotometry. The presence of mutations in the three genes was evaluated by the selective amplification of each gene using PCR. For sequencing, the ABI PRISM® 377 DNA fluorescent sequencer was used.

Based on these findings, a monthly treatment with long-acting GnRH analog (triptorelin) was initiated. After two months, secondary sexual characteristics reverted (Figure 2), and a decrease in the uterine and ovarian volume and suppression of the gonadotropins were observed; the bone age also stabilized.

## 3. Discussion

SFM syndrome associates sebaceous nevus with the involvement of other organs and systems [7]. The other form of nevus that can manifest is the keratinocytic or verrucous nevi [2]. The common anomalies in the different systems are neurological (66%), followed by ophthalmological (59%), skeletal (50%), cardiac (12%), urologic (10%), and endocrinal (<5%) [5]. This case is an atypical one because it presents with verrucous nevi instead of sebaceous nevi. Another frequent characteristic that we could appreciate in the measurement of lower limbs is a hypertrophy of bones of the right half body, ipsilateral to the verrucous nevus, as described in the scientific literature [2]. Another accompanying extracutaneous manifestation is CPP, the suspicion of which was the reason for the endocrine consultation, without evidence of other hormonal abnormalities, such as hypophosphatemic vitamin D-resistant rickets [18, 19]; normal skeletal survey findings; normal levels of calcium, phosphorus, PTH, and vitamin D; and SIADH with normal level of electrolytes and adequate urination.

Mutations in the H-RAS, K-RAS, and N-RAS genes have been described previously in patients with SFM syndrome. Groesser et al. analyzed 65 sebaceous nevi and found a mutation in the H-RAS gene in 95% of the cases and the K-RAS gene in 5% of the cases; mosaicism involving H-RAS c.37G > C and K-RAS c.35G > A has also been described in two patients with SFM syndrome [9]. In our patient, genetic studies revealed a missense mutation in exon 2 of the gene, H-RAS c37G > C (p.G13R), in the skin biopsy sample; however, no mutation was detected in the blood, which confirms the occurrence of somatic mosaicism, probably as a result of a postzygotic mutation. This mutation, p.G13R, was previously described by Hafner et al. in 21 of 24 verrucous nevi with H-RAS gene involvement, reporting it as the most common mutation in a series of 72 verrucous nevi, and the H-RAS (39%) was the most affected of the three RAS genes [10]. This mutation leads to a constitutive activation of MAPK and PI3K-Akt cell signaling pathways, which translates into a higher rate of cell proliferation favoring the development of complex hamartomas in the skin and other organs [9, 11, 13].

There are few reports of SFM syndrome associated with CPP. The first association was reported by Moss et al. [15], in 1991, in a 2-year-4-month-old boy with extensive verrucous nevi and secondary sexual characteristics. The timing of diagnosis and start of treatment was similar to those of our patient; however, the management differed where the treatment in the patient described by Moss et al. involved cyproterone acetate 50–100 mg/day for 3 years; however, when an arrest in the progression of puberty was not evidenced, administration of a long-acting GnRH analog was initiated. This is probably because the GnRH analogs were not commonly used at the time of Moss's case. In our patient, triptorelin was initiated from the time of diagnosis. Similarly, Garg et al. [21] identified a 3-year-old boy with penile growth, pubic hair, increased testicular volume, and advanced bone age, with hyperpigmented verrucous plaques from 3 months of age. He was treated with monthly administration of leuprolide acetate, which is another type of GnRH analog, and a good clinical response was achieved.

Precocious puberty in our patient is due to a premature activation of the hypothalamic-pituitary-gonadal axis. This is confirmed by the hormonal profile, which revealed the levels of the LH and FSH to be in the pubertal range after the GnRH stimulation test. This was supported by advanced bone age and a uterine length and ovarian volume higher than expected for her age on the pelvic ultrasound. In this regard, Ivker et al. [18] hypothesized extensive verrucous nevi release factors that would induce puberty. It is also reasonable to assume that some time is needed for these factors to reach critical concentrations sufficient enough to stimulate the hypothalamic-pituitary axis and lead to the development of secondary sexual characteristics. It is important to perform brain MRI, particularly in cases where the H-RAS gene mutation is present, to rule out hamartomas or other CNS tumors [21]. No type of tumor was evident in our patient.

This case report highlights a fundamental characteristic of epidermal nevus syndromes that have a highly variable systemic involvement. Furthermore, it is important to remember that CPP can develop. Hence, it must be recognized promptly for optimal management, and a multidisciplinary approach must be strategized from the beginning.
